# Mothers and daughters-in-law: a prospective study of informal care-giving arrangements and survival in Japan

**DOI:** 10.1186/1471-2318-10-61

**Published:** 2010-08-29

**Authors:** Akihiro Nishi, Nanako Tamiya, Masayo Kashiwagi, Hideto Takahashi, Mikiya Sato, Ichiro Kawachi

**Affiliations:** 1 Department of Health Services Research, Graduate School of Comprehensive Human Sciences, University of Tsukuba, Ibaraki, Japan; 2 Department of Society, Human Development, and Health, Harvard School of Public Health, Boston, MA, USA; 3 Department of Public Health, Graduate School of Medicine, The University of Tokyo, Tokyo, Japan; 4 Department of Epidemiology and Biostatistics, School of Medicine, University of Tsukuba, Ibaraki, Japan; 5 Tokyo Suginami Centre for Family Medicine, Kawakita General Hospital, Tokyo, Japan

## Abstract

**Background:**

Daughters-in-law have played an important role in informal care-giving arrangements within East Asian traditional norms. The aim of this study was to measure the impact of daughter-in-law care-giving on the survival of care recipients. We prospectively examined the associations between different types of kinship relationship between the main family caregiver and the care recipient in relation to survival among care recipients.

**Methods:**

A questionnaire was administered to Japanese community-dwelling seniors who were eligible to receive national long-term care insurance (LTCI) community-based services. Among 191 individuals whose informal care-giving arrangement was definitively determined, we observed 58 care recipients receiving care from spouses, 58 from daughters-in-law, 27 from biological daughters, 25 from other relatives, and 23 care recipients living alone.

**Results:**

During 51 months of follow-up from December 2001, 68 care recipients died, 117 survived, and 6 moved. Hazard ratios of each care-giving arrangement were estimated by Cox proportional hazard models adjusted for care recipients' demographic factors, their care needs level based on their physical and cognitive functioning and their service use, caregivers' demographic factors, and household size. The highest risk of mortality was found for female elders receiving care from daughters-in-law (HR 4.15, 95% CI 1.02-16.90) followed by those receiving care from biological daughters (HR 1.64, 95% CI 0.37-7.21), compared to women receiving spousal care. By contrast, male elders receiving care from daughters-in-law tended to live longer than those receiving care from their spouses.

**Conclusions:**

Our finding suggests that there may be a survival "penalty" for older Japanese women who are cared for by their daughters-in-law.

## Background

Across societies in East Asia - China, Taiwan, Korea, and Japan -- daughters-in-law have played a central role in providing informal care for older people, in accord with Confucian traditions [1-7]. The traditional life course pattern for Japanese women has been to take care of their parents-in-law and their husbands, then subsequently be cared for by their own daughters-in-law [1]. Although some studies have pointed out that the female caregiver role has changed in Japan due to the increasing popularity of westernized values (including the shift from multi-generational to nuclear family arrangements), and the expanded labor force participation of women, informal care provided by adult children (especially, daughters-in-law living together with their parents-in-law) remains one of the most common sources of care-giving for aging individuals in Japanese society [1,5,6]. According to a national survey, the most common sources of care for Japanese elders are co-habiting spouses (25.9% in 2001, 24.7% in 2004 and 25.0% in 2007), co-habiting children-in-law (22.5% in 2001, 20.3% in 2004 and 14.3% in 2007) and co-habiting biological children (19.9% in 2001, 18.8% in 2004 and 17.9% in 2007)[8-10].

Recent studies have begun to document the potentially deleterious health consequences of intensive care-giving for the health of care providers. For example, Epel and colleagues (2004) [11] demonstrated that women providing care to chronically ill children had significant shortening of leukocyte telomere length - a biological marker of stress and accelerated aging. Other studies have also documented the adverse effects of care-giver stress on markers of immune functioning, such as wound healing and response to influenza virus vaccine [12,13]. Indeed, Schulz & Beach [14] demonstrated that spouse caregivers exhibited a greater risk for mortality than non-caregivers. Although a meta-analysis found that caregivers exhibited a slightly greater risk of health problems compared to non care-givers [15], it has not been conclusively established that providing active help produces harmful levels of stress among caregivers [16]. These studies from western contexts, however, mainly focused on spouses as the main family caregiver, not on daughters-in-law.

In a U.S. prospective study of 54 412 middle-aged women free of heart disease at baseline [17], those who reported providing more than 9 hours of care-giving to sick spouses per week were at nearly twice the risk of developing incident coronary heart disease, compared to women reporting no care-giving duties (Relative risk 1.82, 95% CI 1.08-3.05). In the same cohort study, women who reported providing intensive care to sick parents did not exhibit a similar increased risk of heart disease (multivariable-adjusted RR 0.81, 95% CI 0.43-1.53), which the authors interpreted as the reflection of the fact that the responsibilities for providing care to parents can be shared between siblings, whereas care-giving for sick husbands falls primarily on the shoulders of wives - at least in the U.S. social context.

In contrast to the typical North American pattern of informal care delivery, the primary responsibility for elder care in the East Asian traditional norm has fallen on the shoulders of daughters-in-law, not spouses. This distinctive arrangement has given rise to numerous accounts of mother-in-law/daughter-in-law tensions in the popular literature - e.g. see Niwa Fumio's celebrated novella, "The Hateful Age" (1948). Anecdotes aside, there have been very few empirical attempts to establish the health consequences of care-giving in the Japanese context[18-21]. Emerging studies have sought to examine the association between type of familial living arrangement - as a crude proxy for care-giving responsibilities - and the health status of Japanese women. For example, Ikeda and colleagues [19] prospectively examined the association between living arrangement and risk of coronary heart disease incidence and mortality within a cohort of 90 987 Japanese women and men aged 40-69 years, free of cardiovascular disease at baseline. The authors found that women living in multi-generational households (living with spouse-children-parents; or spouse-parents) had 2.0 to 3.0-fold higher risk of coronary heart disease compared to women living with spouses only. In the same study, Japanese men living in multi-generational households did not experience an increased risk of heart disease compared to those living with their wives only.

In contrast to the foregoing studies which examined the health impact of living arrangement on potential care-givers [14-16], almost no study has examined the effects of different types of care-giving arrangements on the health of the care recipients. Although Merrill [22] suggested that in-law caregivers provide less help to their parents-in-law than blood-tied caregivers, whether these differences translate into different health outcomes for the care recipient remains unknown. Fujino and Matsuda [18] prospectively explored the health impact of 2773 Japanese individuals aged 60 years or older living in Japan, and found that men living with others who were able to provide care to them throughout the day had better survival compared to either men living alone, or living with others who were unable to provide care due to infirmity. By contrast, in the same study, women did not experience the same survival advantage by living with others who were able to provide care for them. The focus of this study, however, was not the relationship of the care-giver to the recipient but rather the former's ability to provide care.

In the present report, we sought to prospectively examine the associations between informal care-giving arrangement (defined by kinship of main family caregiver to the recipient) and care recipients' survival in a cohort of Japanese elders.

## Methods

### Study population

Our study was conducted in a middle-sized city in rural Japan. The population of the city was around 55 000 in 2001 and around 53 000 in 2006. Following the roll-out of the national long-term care insurance (LTCI) program including community-based services and institutional care services in 2000 [23], 871 older people (696 community-dwelling and 175 institutionalized) had been classified as eligible for the LTCI services by 2001 out of approximately 10 000 older people (65 years or older) in the city. In December 2001, the municipality mailed a questionnaire to a 50% random sample (*n *= 348) of all the community-dwelling care recipients (and their primary care-givers) in order to document their needs related to the services and their living arrangement.

Following return of the questionnaires, the municipal authority followed up each care recipient's vital status and service use within the LTCI system data contained in the Vital Statistics of Population and the long-term care insurance claims from the municipality between 1^st ^December 2001 and 28^th ^February 2006 (51 months).

The municipality thus provided the investigators with three sets of data: questionnaire information, vital status, and LTCI service utilization. The Ethics Committee of University of Tsukuba approved the secondary use of all three set of data and any kind of subsequent statistical analyses by the investigators. All data were obtained without any information that could be used to identify each individual.

A total of 216 care recipient/care-giver dyads returned the baseline questionnaire for a response rate of 62.1%. We excluded ten subjects for whom the age of the care recipient was less than 65 years old, because the eligibility criteria of LTCI for people less than 65 years old differs from that for people 65 years or older [24]. We further excluded 15 subjects who did not answer questions about their informal care-giving arrangement (kinship of main family caregiver). Consequently, we utilized the data of 191 care recipients (54.9%) for our study analyses.

### Exposure/covariate assessment

To create the informal care-giving arrangement variable, we utilized information combining the caregiver's gender and kinship relation to the care recipient. Following the classification scheme adopted by the Comprehensive Survey of the Living Conditions of People on Health and Welfare [8], we divided care-giver types into five categories, as follows: a) spousal care (receiving care mainly from co-habiting spouse), b) daughter-in-law care (receiving care mainly from co-habiting daughter-in-law), c) biological daughter care (receiving care mainly from co-habiting biological daughter), d) other relative care (receiving care mainly from other co-habiting relative, such as siblings or grandchildren), and e) living alone.

Other questions asked of the care recipients and their caregivers included demographic information (care recipient's gender and age-group and family caregiver's gender and age-group), and the number of family members living together in the same home. We also recorded the care needs level for each individual at the beginning of the follow-up. All care recipients were classified into six care needs levels, ranging from assistance required to care needs levels 1 through 5, based upon each individual's level of physical and cognitive functioning[24]. The utilization of five types of LTCI services are shown: daycare service (nursing home daycare and health-related daycare), home-help service (home help with care-giving or housekeeping), home-visit nursing, respite stay (short-term nursing home stay), and institutional care (special nursing home, health service facility for the elderly, and sanatorium type medical care facility for the elderly requiring care for a long-term period). All of the five variables were given a dichotomous number based upon whether care recipients had utilized each LTCI service for at least one month during the follow-up. We selected the one-month cutoff because it is the shortest duration of service use that certified care managers (care planners) are able to assign to the care recipient - and hence likely to be the most sensitive measure of service use [24,25]. Table 1 shows the basic characteristics in December 2001 and the patterns of LTCI service use in our cohort from 1^st ^December 2001 to 28^th ^February 2006.

**Table 1:** Baseline characteristics, formal service use, and survival of community-dwelling elders in a city in Japan

		Informal care-giving arrangement (kinship of main family caregiver)				
		(main two categories)		(other three categories)		
	All	Spousal care	Daughter-in-law care	Biological daughter care	Other relative care	Living alone
	n (%)	n (%)	n (%)	n (%)	n (%)	n (%)
	191 (100	58 (100.0)	58 (100.0)	27 (100.0)	25 (100.0)	23 (100.0)
Baseline characteristics						
Care recipient's gender						
Female	129 (67.5)	19 (32.8)	48 (82.8)	24 (88.9)	20 (80.0)	18 (78.3)
Male	62 (32.5)	39 (67.2)	10 (17.2)	3 (11.1)	5 (20.0)	5 (21.7)
Care recipient's age-group (year)						
65-79	80 (41.9)	38 (65.5)	20 (34.5)	4 (14.8)	11 (44.0)	7 (30.4)
80-89	81 (42.4)	18 (31.0)	23 (39.7)	18 (66.7)	9 (36.0)	13 (56.5)
90-	29 (15.2)	2 (3.5)	15 (25.9)	5 (18.5)	4 (16.0)	3 (13.0)
Baseline care needs level						
Assistance required	24 (12.6)	2 (3.5)	7 (12.1)	5 (18.5)	4 (16.0)	6 (26.1)
Care needs level 1 and 2	105 (55.0)	34 (58.6)	32 (55.2)	15 (55.6)	11 (44.0)	13 (56.5)
Care needs level 3, 4 and 5	50 (26.2)	18 (31.0)	18 (31.0)	7 (25.9)	5 (20.0)	2 (8.7)
Number of people living together						
0-5	153 (80.1)	46 (79.3)	39 (67.4)	24 (88.9)	22 (88.0)	22 (95.7)
6-	35 (18.3)	11 (19.0)	19 (32.8)	3 (11.1)	2 (8.0)	0 (0.0)
Care-giver's age-group (year)*						
-49	37 (19.4)	1 (1.7)	21 (36.2)	7 (25.9)	8 (40.0)	-
50-59	38 (19.9)	2 (3.5)	18 (31.0)	12 (44.4)	6 (30.0)	-
60-69	40 (20.9)	17 (29.3)	12 (20.7)	7 (25.9)	4 (20.0)	-
70-	47 (24.6)	38 (65.5)	6 (10.3)	1 (3.7)	2 (10.0)	-
Formal service use in follow-up						
Daycare service use						
No	46 (24.1)	19 (32.8)	6 (10.3)	4 (14.8)	10 (40.0)	7 (30.4)
Yes	145 (75.9)	39 (67.4)	52 (89.7)	23 (85.2)	15 (60.0)	16 (69.6)
Home help service use						
No	86 (45.0)	25 (43.1)	31 (53.5)	15 (55.6)	11 (44.0)	4 (17.4)
Yes	105 (55.0)	33 (56.9)	27 (46.6)	12 (44.4)	14 (56.0)	19 (82.6)
Home-visit nursing service use						
No	151 (79.1)	45 (77.6)	46 (79.3)	18 (66.7)	22 (88.0)	20 (87.0)
Yes	40 (20.9)	13 (22.4)	12 (20.7)	9 (33.3)	3 (12.0)	3 (13.0)
Respite stay service use						
No	139 (72.8)	39 (67.2)	40 (69.0)	16 (59.3)	23 (92.0)	21 (91.3)
Yes	52 (27.2)	19 (32.8)	18 (31.3)	11 (40.7)	2 (8.0)	2 (8.7)
Institutional care service use						
No	131 (68.6)	33 (56.9)	47 (81.3)	16 (59.3)	18 (72.0)	17 (73.9)
Yes	60 (31.4)	25 (43.1)	11 (19.0)	11 (40.7)	7 (28.0)	6 (26.1)
						
Cumulative survival rate	(%)	(%)	(%)	(%)	(%)	(%)
All						
After 24-month follow-up	79.4	77.6	74.0	88.9	83.3	82.6
After 48-month follow-up	66.0	63.5	56.4	74.1	70.8	82.6
Male						
After 24-month follow-up	72.6	71.8	80.0	100.0	60.0	60.0
After 48-month follow-up	57.6	53.2	80.0	66.7	40.0	60.0
Female						
After 24-month follow-up	82.7	89.5	72.7	87.5	89.5	88.9
After 48-month follow-up	70.0	84.2	51.3	75.0	79.0	88.9

* The 29 missing data included all the 23 older people living alone.

The outcome of our study was care recipient's survival time. Survival time was monitored during the follow-up period, from 1^st ^December 2001 to 28^th ^February 2006. When individuals died or moved during follow-up, the date of death or the move was recorded. For each individual, length of follow-up period was calculated from 1^st ^December 2001 until the date of death or move from the municipality. Otherwise, individuals were proved to be alive as of 28^th ^February 2006 and recognized as censored cases.

### Statistical analysis

We estimated cumulative survival rates 24-months and 48-months after the beginning of follow-up on 1st December 2001 according to the five categories of informal care-giving arrangement for males, females and both genders combined. The difference between the main two categories: spousal care and daughter-in-law care was tested by log-rank statistic, utilizing the Kaplan-Meier method. Because of the small sample size for males, we merged the following categories: receiving biological daughter care, receiving other relative care, and living alone. We checked the proportionality of the Cox proportional hazard models graphically by using the negative log plots of the survivor function, and the interactions among gender and informal care-giving arrangement were explored by Cox models. We then estimated the effect of informal care-giving arrangement for female care recipient's survival by Cox models. A two-tailed p value of less than 0.05 was considered statistically significant. We carried out all the analyses using SAS version 9.1.3.

## Results

Of the 191 care recipients whom the municipality followed for 51 months, 68 died, 6 moved (and were lost to follow-up), and 117 survived until the study's end, yielding a total of 631.3 person-years of follow-up (188.5 for men and 442.8 for women). Table 1 shows the cumulative survival rates up to 24-months and 48-months following the start of follow-up for male and female care recipients.

In the overall sample, there were no significant differences in survival among the care-giving arrangement categories (log rank, p = 0.252) nor in the comparison between care recipients receiving spousal care versus those receiving daughter-in-law care (log rank, p = 0.430). However, this overall finding obscured a significant gender difference in the pattern of survival according to source of care.

**Figure 1:**
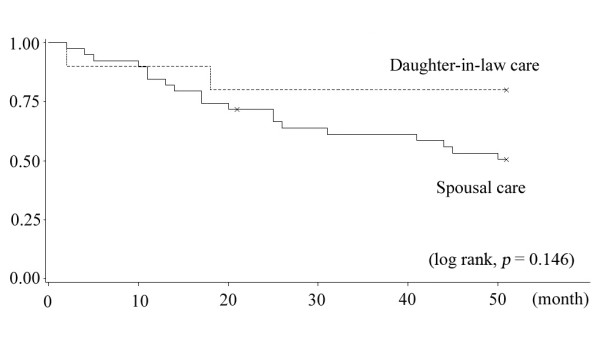
**Kaplan-Meier curves of male care recipients between spousal care and daughters-in-law**.

**Figure 2:**
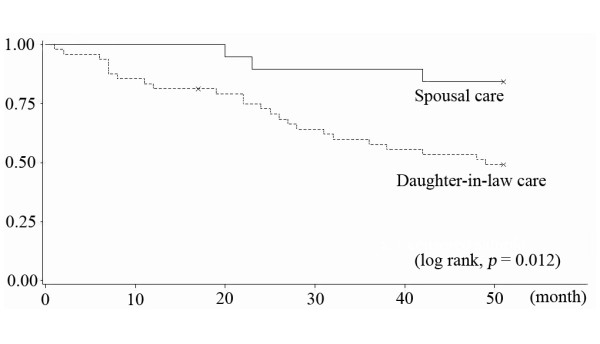
**Kaplan-Meier curves of female care recipients between spousal care and daughters-in-law**.

Figure 1 shows that male elders receiving care from daughters-in-law had better survival compared to those receiving spousal care (log rank, p = 0.146). By stark contrast, among women in our sample, care recipients receiving spousal care had better survival than care recipients receiving daughter-in-law care (log rank, p = 0.012) (Figure 2).

Table 2 explored the interaction of the two variables: care recipient's gender and care-giving arrangement. Both male gender (p = 0.027) and daughter-in-law care are (p = 0.022) risk factors for increased mortality. By contrast, the coefficient of the product term (male care recipient receiving care from a daughter-in-law) was negative (p = 0.011). This can suggest that the effect of daughter-in-law care for survival among male care recipients was protective, as opposed to the pattern among female care recipients. The magnitude of the interaction, however, diminished after adjustment for other covariates in Model II (p = 0.134).

**Table 2:** Interaction between care recipient's gender and care-giving arrangement in Cox proportional hazard models

	Model I*		Model II**	
Variable names	HR (95% CI)	P	HR (95% CI)	P
Care recipient's gender				
Female	1.00		1.00	
Male	3.97 (1.17 - 13.42)	0.027	5.13 (2.25 - 11.69)	< 0.0001
				
Informal care-giving arrangement				
Spousal care	1.00		1.00	
Daughter-in-law care	4.09 (1.23 - 13.58)	0.022	4.40 (1.60 - 12.10)	0.004
Other categories (biological daughter care/other relative care/living alone)	1.55 (0.45 - 5.39)	0.491	1.26 (0.55 - 2.90)	0.580
				
Interaction				
Male gender * Daughter-in-law care	0.09 (0.01 - 0.57)	0.011	0.08 (0.02 - 0.46)	0.004
Male gender * Other categories	0.66 (0.14 - 3.11)	0.601		

* No adjusting variables in this model.

** Adjusting care recipient's age, baseline care needs level, number of people living together, daycare service use, home-help servie use, home-visit nursing service use, home-visit nursing use, respite stay, and institutional care use

Gender-specific stratified multivariable analysis was carried out among women care recipients but not among males because of small sample size. Table 3 Model III and IV shows the results of the regression models among female care recipients. Even after controlling for the possible confounding factors (Model IV), women who received care from daughters-in-law were 4.3 times more likely to die during the follow-up period, compared to women receiving care from their spouses. In addition, those who received care from biological daughters were approximately 1.88 times more likely to die, compared to the same reference, but not significant.

**Table 3:** Hazard Ratios of informal and formal setting in female care recipients in Cox proportional hazard models

	Model III (without adjustment)*		Model IV (with adjustment)	
	HR	95% CI	HR	95% CI
Informal care-giving arrangement				
Spousal care	1.00		1.00	
Daughter-in-law care	4.14	(1.25 - 13.77)	4.15	(1.02 - 16.90)
Biological daughter care	1.97	(0.51 - 7.62)	1.64	(0.37 - 7.21)
Other relative care	1.76	(0.42 - 7.35)	1.85	(0.35 - 9.94)
Living alone	0.76	(0.13 - 4.53)	0.46	(0.04 - 5.48)
Care recipient's age (continuous)	1.04	(1.00 - 1.09)	1.02	(0.98 - 1.08)
Baseline care needs level				
Assistance required	1.00		1.00	
Care needs level 1	1.26	(0.40 - 3.95)	1.41	(0.42 - 4.75)
Care needs level 2	1.69	(0.49 - 5.77)	1.45	(0.40 - 5.30)
Care needs level 3	2.80	(0.84 - 9.32)	3.25	(0.92 - 11.46)
Care needs level 4 or 5	3.18	(0.96 - 10.59)	3.21	(0.81 - 12.74)
Number of people living together (continuous)	1.18	(1.01 - 1.38)	0.99	(0.78 - 1.25)
Daycare service use				
No	1.00		1.00	
Yes	0.71	(0.36 - 1.39)	0.51	(0.22 - 1.22)
Home help service use				
No	1.00		1.00	
Yes	0.61	(0.33 - 1.13)	0.64	(0.31 - 1.32)
Home-visit nursing service use				
No	1.00		1.00	
Yes	1.09	(0.54 - 2.23)	0.71	(0.28 - 1.80)
Respite stay service use				
No	1.00		1.00	
Yes	0.95	(0.47 - 1.89)	0.86	(0.38 - 1.94)
Institutional care service use				
No	1.00		1.00	
Yes	0.63	(0.31 - 1.29)	0.88	(0.38 - 2.05)

* Each HR was obtained separately variable by variable.

## Discussion

To our knowledge, this is the first study to examine the associations between kinship of the primary caregiver and survival of care recipients in the Japanese context. The findings are provocative in that they suggest a survival disadvantage for women who are cared for by their daughters-in-law, i.e. a daughter-in-law "penalty."

Potential explanations for the daughter-in-law penalty remain unclear. However, two possible hypotheses to account for the increased risk of mortality among women receiving care from their daughters-in-law are: a) strained emotional relationships between mothers-in-law and daughters-in-law resulting in caregivers' psychological abuse and consequent neglect of care, and/or b) the care recipients' own mental state, which may range from depression to self-neglect.

According to research on elder abuse carried out by Soeda & Araki [26], major perpetrators in Japan tend to be daughters-in-law (30 to 40%). They also found that the most frequent reason given for abuse by daughters-in-law was a poor relationship with their in-laws (60.0%), followed by stress (40.0%). A study by Institute for Health Economics and Policy (2003) found that daughters-in-law were more frequently identified as perpetrators of neglect (62.8%) and psychological abuse (72.7%) compared to physical abuse (34.5%) or financial abuse (17.0%). In turn, mistreatment such as neglect and psychological abuse is one of the major causes of care recipients' death as pointed in an American study, which reported that older people who were mistreated were 3.1 times more likely to die during a 3-year period than those who did not experience abuse [27].

What account for the strained relations between mothers-in-law and daughters-in-law? Indeed, Koyano and colleagues [28] found that biological children were more likely to have closer and better relationships with aging parents than children-in-law. Three characteristics of long-term poor relationships were also described as: a) living with parents-in-law, who are not related, come from a different generation, and have a different life-style causes a lot of stress, b) sons who fail to mediate the relationship between their wives and their parents, and c) mothers-in-law who tend to interfere in their sons' family because of their intimate feelings toward their sons [26]. Their study also pointed that relationships between daughters-in-law and mothers-in-law are often much worse than relationships between daughters-in-law and fathers-in-law, because daughters-in-law and mothers-in-law have a lot of disputes over the authority to make decisions regarding housework in a Japanese traditional family (a son and his wife living with his parents in the same household).

Some researchers reported the possibility of underutilization of social services under the Japanese LTCI system due to social norms and the opinions of family caregivers [29-31]. Japan introduced legislation addressing elder abuse prevention and caregiver support in 2007 [31], and these initiatives may help to support families with fragile informal care-giving arrangements [30,32].

It is possible that our main findings, including the "daughter-in-law penalty", might reflect confounding by underlying common causes - for example, daughters-in-law may be selectively recruited into providing care for sicker relatives. Additionally, in our study, we did not control for comorbidity or other measures of health status. However, our multivariable analyses did control for baseline differences in the care needs level of the recipients. On the other hand, since the potential unobserved confounders (such as comorbidity) would affect the care-giver arrangement through the "care needs level" variable, we could still obtain an unbiased estimate of the effect of informal care-giving arrangement on mortality by controlling for the latter variable.

We added institutional care into the multivariable analyses as one of the control variables (Model II and Model IV), because the effect of informal care-giving arrangement could theoretically persist even after the care recipient became institutionalized. For example, there is a potential latency/induction period between receipt of care and mortality, which may be months to several years. As an extra analysis complying with another interpretation that there is no latency/induction period, we dealt with the care recipients who were institutionalized as censored at the moment of being institutionalized in addition to the care recipients who were moved out from the municipality. The adjusted HR of mortality among those receiving daughter-in-law care was 3.31 (95% CI 0.61-18.04), and 1.68 (95% CI 0.24-11.55) for those cared by the biological daughter. In other words, our findings were similar to the HRs in Table 3 (Model IV without control for institutional care).

The other possible limitations need to be noted when interpreting the results of our study. Our study is not nationally representative, and hence our findings may not be wholly generalizable to Japanese society. There is also the possibility of selection bias because of the response rate to the baseline questionnaire. At baseline, however, regarding the response rate on informal care-giving arrangement (overall response rate; 54.9%, 191/348), there were no major differences by gender (response rate; male: 51.2% and female: 56.8%) or care needs level (response rate; assistance required: 50.0%, care needs level 1-2: 55.0% and care needs level 3-5: 47.6%).

Some observers have linked the ongoing decline in marital fertility in Japanese society to the evolving care-giving crisis in that same country [6,33]. During the past two decades, in tandem with the increased aging population, there has been a steady rise in the age at marriage for Japanese women and men, accompanied by an attendant collapse in the fertility rate, which is among the lowest in the world: 1.37 (total fertility rate in 2008) [34]. These trends have been reflected by a popular saying on the streets of Japan, which advises young women to choose their husbands carefully - preferably, sons whose parents have already passed away!

## Conclusion

In summary, our study suggests a survival "penalty" for older women cared for by daughters-in-law. Our findings require replication in additional studies, as well as further research to elucidate the underlying mechanisms. If our findings are corroborated, there are two major implications. First, it would suggest that the longevity of Japanese women (currently the highest in the world) is unlikely to be explained by the traditional arrangement of inter-generational care-giving, mainly had been performed by daughter-in-law, toward the end of life. Second, our findings suggest that reliance upon traditional, informal care-giving arrangements may not be the optimal solution to dealing with the aging of Japanese society, and that policy approaches are needed to support the care-giving needs of a rapidly aging population.

## Competing interests

The authors declare that they have no competing interests.

## Authors' contributions

AN carried out structuring the study design, statistical analysis, interpreting the data, and drafting the manuscript. NT supervised all the process as the corresponding author: participated in the design of the study, statistical analysis, interpretation of the data, and helped to finalize the manuscript. MK participated in designing this study, acquiring the data, and structuring the data set. HT helped to create the SAS program to perform the statistical analysis and supervised statistical process. MS carried out special advice to structure the data set. IK participated in interpretation of the data and helped to finalize the manuscript. All authors read and approved the final manuscript.

## Pre-publication history

The pre-publication history for this paper can be accessed here:

http://www.biomedcentral.com/1471-2318/10/61/prepub

## References

[B1] Hashizume Y Gender issues and Japanese family-centered caregiving for frail elderly parents or parents-in-law in modern Japan From the sociocultural and historical perspectives Public Health Nursing 2000 17 25 31 10.1046/j.1525-1446.2000.00025.x 10675050

[B2] Kao H McHugh M The role of caregiver gender and caregiver burden in nursing home placements for elderly Taiwanese survivors of stroke Res Nurs Health 2004 27 121 134 10.1002/nur.20007 15042638

[B3] Kim JS Daughters-in-law in Korean caregiving families J Adv Nurs 2001 36 399 408 10.1046/j.1365-2648.2001.01987.x 11686754

[B4] Koh EK Koh CK Caring for older adults - The parables in confucian texts Nursing Science Quarterly 2008 21 365 368 10.1177/0894318408324320 18953016

[B5] Long SO Harris PB Gender and elder care: social change and the role of the caregiver in Japan Social Science Japan Journal 2000 3 21 36 10.1093/ssjj/3.1.21

[B6] Tsuji I Sauvaget C Hisamichi S Health expectancies in Japan gender differences and policy implications for women Journal of Women & Aging 2002 14 135 148 10.1300/J074v14n01_09 12537284

[B7] Yamamoto N Wallhagen MI The continuation of family caregiving in Japan Journal of Health and Social Behavior 1997 38 164 176 10.2307/2955423 9212537

[B8] Statistics and Information Department Minister's secretariat, Ministry of Health, Labour and Welfare (MHLW) (Japan) Comprehensive survey of Living Conditions of the People on Health and Welfare 2001 (in Japanese)

[B9] Statistics and Information Department Minister's secretariat, Ministry of Health, Labour and Welfare (MHLW) (Japan) Comprehensive survey of Living Conditions of the People on Health and Welfare 2004 (in Japanese)

[B10] Statistics and Information Department Minister's secretariat, Ministry of Health, Labour and Welfare (MHLW) (Japan) Comprehensive survey of Living Conditions of the People on Health and Welfare 2007 (in Japanese)

[B11] Epel ES Blackburn EH Lin J Dhabhar FS Adler NE Morrow JD Cawthon RM Accelerated telomere shortening in response to life stress Proc Natl Acad Sci USA 2004 101 17312 17315 10.1073/pnas.0407162101 15574496 PMC534658

[B12] Kiecoltglaser JK Marucha PT Malarkey WB Mercado AM Glaser R Slowing of wound-healing by psychological stress Lancet 1995 346 1194 1196 10.1016/S0140-6736(95)92899-5 7475659

[B13] KiecoltGlaser JK Glaser R Gravenstein S Malarkey WB Sheridan J Chronic stress alters the immune response to influenza virus vaccine in older adults Proc Natl Acad Sci USA 1996 93 3043 3047 10.1073/pnas.93.7.3043 8610165 PMC39758

[B14] Schulz R Beach SR Caregiving as a risk factor for mortality - The caregiver health effects study JAMA-J Am Med Assoc 1999 282 2215 2219 10.1001/jama.282.23.2215 10605972

[B15] Vitaliano PP Zhang JP Scanlan JM Is caregiving hazardous to one's physical health? A meta-analysis Psychol Bull 2003 129 946 972 10.1037/0033-2909.129.6.946 14599289

[B16] Brown SL Smith DM Schulz R Kabeto MU Ubel PA Poulin M Yi J Kim C Langa KM Caregiving Behavior Is Associated With Decreased Mortality Risk Psychological Science 2009 20 488 494 10.1111/j.1467-9280.2009.02323.x 19320860 PMC2865652

[B17] Lee S Colditz GA Berkman LF Kawachi I Caregiving and risk of coronary heart disease in US women - A prospective study American Journal of Preventive Medicine 2003 24 113 119 10.1016/S0749-3797(02)00582-2 12568816

[B18] Fujino Y Matsuda S Prospective study of living arrangement by the ability to receive informal care and survival among Japanese elderly Preventive Medicine 2009 48 79 85 10.1016/j.ypmed.2008.10.014 19010348

[B19] Ikeda A Iso H Kawachi I Yamagishi K Inoue M Tsugane S for the JPHC Study Group Living arrangement and coronary heart disease: the JPHC study Heart (London) 2009 95 577 583 10.1136/hrt.2008.149575 19066191

[B20] Liang J Shaw BA Krause NM Bennett JM Blaum C Kobayashi E Fukaya T Sugihara Y Sugisawa H Changes in functional status among older adults in Japan: Successful and usual aging Psychology and Aging 2003 18 684 695 10.1037/0882-7974.18.4.684 14692857

[B21] Sato T Kishi R Suzukawa A Horikawa N Saijo Y Yoshioka E Effects of social relationships on mortality of the elderly: How do the influences change with the passage of time? Archives of Gerontology and Geriatrics 2008 47 327 339 10.1016/j.archger.2007.08.015 17936379

[B22] Merrill DM Daughter-in-law as caregiviers to the elderly - Defining the in-law relationship Research on Aging 1993 15 70 91 10.1177/0164027593151004

[B23] Campbell JC Ikegami N Long-term care insurance comes to Japan Health Affairs 2000 19 26 39 10.1377/hlthaff.19.3.26 10812779

[B24] Tsutsui T Muramatsu N Care-needs certification in the long-term care insurance system of Japan J Am Geriatr Soc 2005 53 522 527 10.1111/j.1532-5415.2005.53175.x 15743300

[B25] Asahara K Momose Y Murashima S Long-tern care insurance in Japan: Its frameworks, issues, and roles Dis Manage Health Outcomes 2003 11 769 777 10.2165/00115677-200311120-00002

[B26] Soeda A Araki C Elder abuse by daughters-in-law in Japan Journal of Elder Abouse & Neglect 1999 11 47 58

[B27] Lachs MS Williams CS O'Brien S Pillemer KA Charlson ME The mortality of elder mistreatment JAMA-J Am Med Assoc 1998 280 428 432 10.1001/jama.280.5.428 9701077

[B28] Koyano W Okamura K Ando T Hasegawa M Asakawa T Kodama Y Characteristics of children affecting the relationships between elderly parents and their children Japanese Journal of Gerontology 1995 16 136 145

[B29] Asai MO Kameoka VA The influence of Sekentei on family caregiving and underutilization of social services among Japanese caregivers Social Work 2005 50 111 118 15853188 10.1093/sw/50.2.111

[B30] Tamiya N Yamaoka K Yano E Use of home health services covered by new public long-term care insurance in Japan: impact of the presence and kinship of family caregivers International Journal for Quality in Health Care 2002 14 295 303 10.1093/intqhc/14.4.295 12201188

[B31] Nakanishi M Hoshishiba Y Iwama N Okada T Kato E Takahashi H Impact of the elder abuse prevention and caregiver support law on system development among municipal governments in Japan Health Policy 2009 90 254 261 10.1016/j.healthpol.2008.10.009 19041151

[B32] Hanaoka C Norton EC Informal and formal care for elderly persons How adult children's characteristics affect the use of formal care in Japan Social Science & Medicine 2008 67 1002 1008 10.1016/j.socscimed.2008.05.006 18579273

[B33] Takeda Y Kawachi I Yamagata Z Hashimoto S Matsumura Y Oguri S Okayama A Multigenerational family structure in Japanese society: inpacts on stress and health behaviours among women and men Social Science & Medicine 2004 59 69 81 10.1016/j.socscimed.2003.10.003 15087144

[B34] Statistics and Information Department Minister's secretariat, Ministry of Health, Labour and Welfare (MHLW) (Japan) Vital Statistics 2008 (in Japanese)

